# Information filtering by coincidence detection of synchronous population output: analytical approaches to the coherence function of a two-stage neural system

**DOI:** 10.1007/s00422-020-00838-6

**Published:** 2020-06-24

**Authors:** Žiga Bostner, Gregory Knoll, Benjamin Lindner

**Affiliations:** 1 Physics Department, Humboldt University Berlin, Newtonstr. 15, 12489 Berlin, Germany; 2 Bernstein Center for Computational Neuroscience Berlin, Philippstr. 13, Haus 2, 10115 Berlin, Germany

**Keywords:** Information coding, Synchronization, Coincidence detection, Neural computation

## Abstract

Information about time-dependent sensory stimuli is encoded in the activity of neural populations; distinct aspects of the stimulus are read out by different types of neurons: while overall information is perceived by integrator cells, so-called *coincidence detector* cells are driven mainly by the synchronous activity in the population that encodes predominantly high-frequency content of the input signal (high-pass information filtering). Previously, an analytically accessible statistic called the partial synchronous output was introduced as a proxy for the coincidence detector cell’s output in order to approximate its information transmission. In the first part of the current paper, we compare the information filtering properties (specifically, the coherence function) of this proxy to those of a simple coincidence detector neuron. We show that the latter’s coherence function can indeed be well-approximated by the partial synchronous output with a time scale and threshold criterion that are related approximately linearly to the membrane time constant and firing threshold of the coincidence detector cell. In the second part of the paper, we propose an alternative theory for the spectral measures (including the coherence) of the coincidence detector cell that combines linear-response theory for shot-noise driven integrate-and-fire neurons with a novel perturbation ansatz for the spectra of spike-trains driven by colored noise. We demonstrate how the variability of the synaptic weights for connections from the population to the coincidence detector can shape the information transmission of the entire two-stage system.

## Introduction

How complex, time-dependent signals are encoded in the stochastic spike trains of sensory neurons is an important problem in computational neuroscience. Claude Shannon’s theory of communication (Shannon 1948) offers a mathematical framework to quantify the amount of information that a spike train encodes about a sensory stimulus (Rieke et al. 1996; Borst and Theunissen 1999). With a measure of encoded information at hand (e.g., the spectral coherence function (Borst and Theunissen 1999) or the frequency-resolved mutual information (Bernardi and Lindner 2015)) and specifically for a broadband stimulus, we can furthermore ask how much information is transmitted in the different frequency bands, i.e., how much information about slow, intermediate or fast stimulus components the spike train contains. This question has been studied for different sensory modalities, for instance, for vision (Warland et al. 1997; Reinagel et al. 1999; Passaglia and Troy 2004), in the auditory (Rieke et al. 1995; Marsat and Pollack 2004) and vestibular (Sadeghi et al. 2007; Massot et al. 2011) systems, and in the electrosensory systems of weakly electric fish (Chacron et al. 2003; Oswald et al. 2004; Chacron 2006; Middleton et al. 2009), and paddle fish (Neiman and Russell 2011).

The concept of *information filtering* [see the review by Lindner (2016)] has been useful to understand the potential functional role of certain features seen in the spontaneous firing of neurons. For instance, pronounced negative ISI correlations in the spontaneous activity of a nerve cell, as observed in electrosensory cells in weakly electric fish (Ratnam and Nelson 2000; Chacron et al. 2000), can drastically enhance the transmission of low-frequency stimuli relevant for the animal (Chacron et al. 2001, 2004) [for reviews on ISI correlations and their role in neural signal transmission, see Farkhooi et al. (2009), Avila-Akerberg and Chacron (2011)]. Temporally correlated (“colored”) noise, e.g., resulting from adaptation channels (Fisch et al. 2012), may lead to positive ISI correlations and thus suppress information transmission at low frequencies (Blankenburg and Lindner 2016) (band-pass information filtering). The same band-pass shaping has been shown by Droste and Lindner (2017c) for signal transmission in the presence of colored two-state fluctuations (telegraph noise) that result from up-and-down states at the network level (Steriade et al. 1993; Cowan and Wilson 1994). Subthreshold resonances in neural dynamics, as observed in the broad class of resonator neurons (Izhikevich 2001; Brunel et al. 2003; Izhikevich 2007), lead to a band-pass filter centered around the resonance frequency (Blankenburg et al. 2015). For bursting cells, it has been shown that different components of the output (single spikes vs bursts of spikes) can encode information from distinct stimulus frequency bands (Oswald et al. 2004), a form of parallel processing that relies on information filtering. Last but not least, synaptic dynamics such as short-term synaptic depression and facilitation may shape the information filter (Lindner et al. 2009; Merkel and Lindner 2010; Rosenbaum et al. 2012; Droste et al. 2013).

Most relevant to the subject of this paper is the information filtering observed at the population level: If a neural population of uncoupled cells is driven by a common broadband stimulus, their summed output encodes slow stimulus components best (Middleton et al. 2009; Beiran et al. 2017), but if we focus on the spikes that are jointly fired in the population, this synchronous output preferentially encodes stimulus components from an intermediate frequency band (Middleton et al. 2009). The latter effect can be understood by an analysis of the spectral statistics of the so-called partial synchronous output (Sharafi et al. 2013; Kruscha and Lindner 2016; Kruscha 2017). A recent study has highlighted the importance of the intrinsic noise level and the strength of the leak current by comparing two types of sensory cells that differ considerably in these properties and, consequently, also differ in the information filtering properties of their synchronous outputs (Grewe et al. 2017). We mention in passing that, generally, connection topology also shapes information filtering at the network level, see, e.g., Åkerberg and Chacron (2009), Deger et al. (2014).

**Fig. 1:**
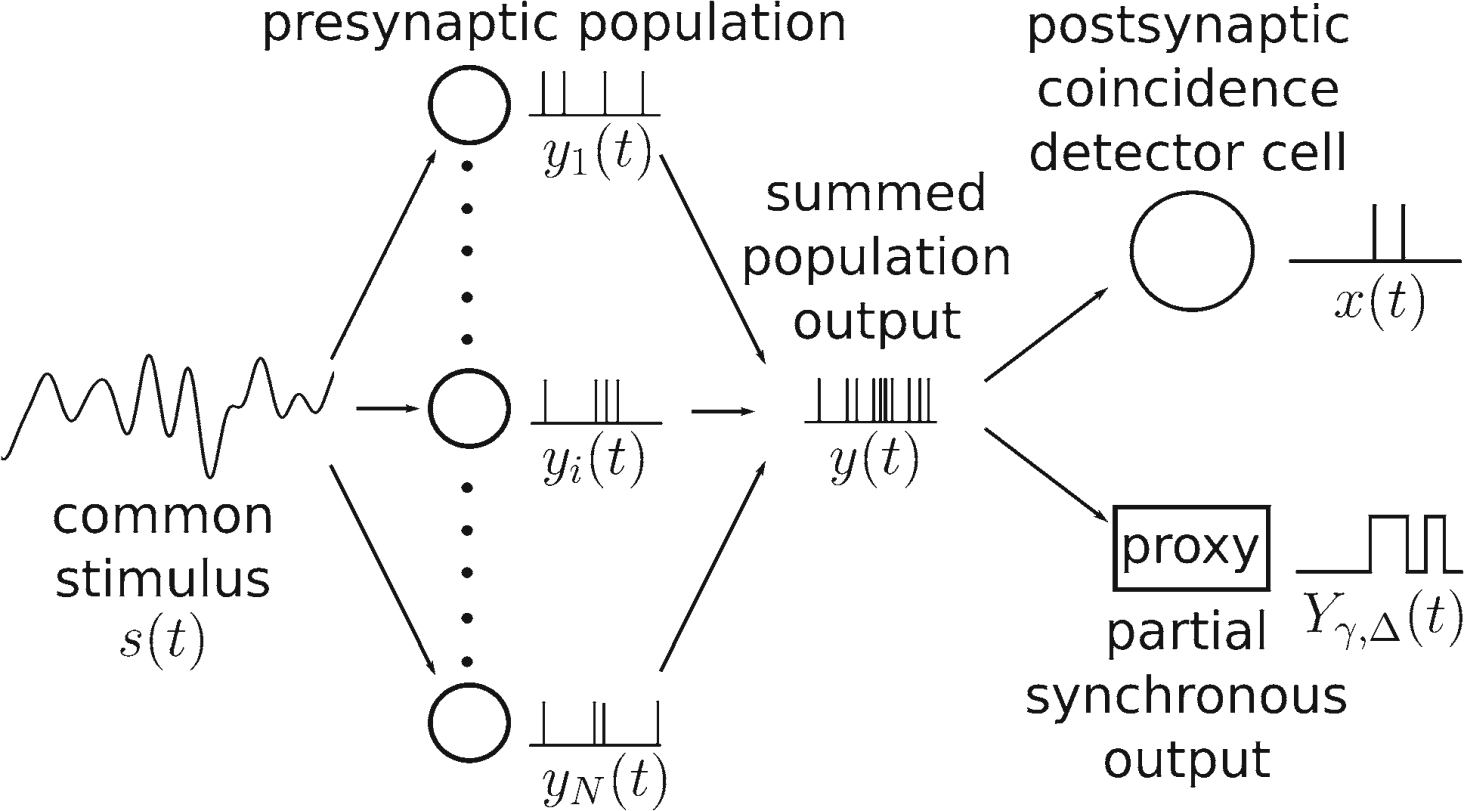
Schematic illustration of the model. In the first stage, the presynaptic population is driven in part by a weak common stimulus, *s*(*t*), and otherwise by independent intrinsic noise unique to each neuron. In the second stage, the summed output of the population, *y*(*t*), acts as an input either for the leaky integrate-and-fire model of the coincidence detector cell (last stage, top) or for the partial synchronous output (last stage, bottom). The output of the CD is a spike train *x*(*t*), whereas the SO output is a two-state time series $Y_{\gamma , \varDelta }(t)$. The parameters of the presynaptic population remain fixed throughout the paper, unless otherwise noted: $N=100$, $\tau _\mathrm{{POP}}=1$, $\mu _\mathrm{{POP}}=1.2$, $D=0.01$, $c=0.1$

Studying the filtering properties of the synchronous output of a population is certainly interesting in its own right: the concept of synchrony has been well established throughout science (Pikovsky et al. 2001) and is especially important in neuroscience in the contexts of information transmission (Dan et al. 1998; Reyes 2003), attention (Tiesinga et al. 2004), and the binding hypothesis (Singer 1999; Shadlen and Movshon 1999). Synchrony can be easily extracted from multi-electrode recordings (Schneidman et al. 2006; Shlens et al. 2006; Kreiter and Singer 1996) and is also amenable to analytical approaches (Sharafi et al. 2013; Kruscha and Lindner 2016; Kruscha 2017). However, in a real system synchronous activity has to be read out in some way by neurons at a second stage of processing (König et al. 1996): a coincidence detector cell that will be activated only by a volley of spikes, i.e., by synchronous activity of the population. The extent to which the information transmission by the synchronous output agrees with that of a coincidence detector cell has not yet been investigated systematically. This problem is obviously relevant for the question of whether we can substitute one (the synchronous output) for the other (the coincidence detector cell’s output), and it is addressed for the simple model system of Fig. 1 in the first part of our paper.

Analyzing how population synchrony encodes time-dependent signals thus gives us an approximate idea how second-stage neurons in a coincidence detector mode would encode these signals. There is, however, also a need for alternative methods to calculate the information flow over several stages of neural processing. In the second part of the paper, we develop an approximation for the spectral measures of the simple two-stage system in Fig. 1, in particular the coherence function which characterizes the information transmission of the system as a whole. We compare all analytical results to numerical simulations of the stochastic population model and show that our approximation works well for physiologically reasonable parameters. In the context of information filtering, we highlight a source of high-pass filtering that has so far been overlooked: the unreliability of synaptic amplitudes that is frequently observed experimentally (Lefort et al. 2009) may contribute to a suppression of the information flow in the low-frequency band. As a consequence, even if the postsynaptic cell is *not* in a coincidence-detector mode, it may still encode most information about stimulus components from an intermediate frequency band, the mechanism of which can be understood in terms of some simple analytical estimates.

Our paper is organized as follows. We present the model and the measures of interest in the next section. The relation between the partial synchronous output and the spikes of the second-stage coincidence detector cell is addressed in Sect. 3. In Sect. 4, we outline our analytical approximation for the spectral coherence function of the second-stage neuron (further details are given in appendix Sect. 1) and discuss the role synaptic weights play in information filtering between the stages. We conclude the paper with a brief summary of our main results and a discussion of possible extensions of the theory.

## Model and measures of interest

### Presynaptic population (first stage)

The presynaptic population consists of *N* uncoupled nerve cells, which are modeled by leaky integrate-and-fire (LIF) neurons and driven by broadband Gaussian noise (see Fig. 1, left). The voltage dynamics of the *i*-th neuron are governed by

1 $$\begin{aligned} \tau _\mathrm{{POP}} ~ \dot{v}_i = - v_i + \mu _\mathrm{{POP}} + s(t) + \sqrt{2(1-c)D}\xi _i(t) \end{aligned}$$

in addition to the fire-and-reset rule, which dictates that the voltage $v_i$, upon reaching the threshold voltage $v_{T, POP}=1$, is set to the reset voltage $v_{r, POP}=0$. Reaching the threshold represents a neuronal spike and the times $t_j$ of all such events are recorded and the time series of spikes for the *i*-th neuron is then expressed as $y_i(t)=\sum _j \delta (t - t_{i,j})$. In what follows, we measure time in multiples of the membrane time constant $\tau _\mathrm{{POP}}$ (and rates and frequencies in multiples of its inverse) and thus set $\tau _\mathrm{{POP}}=1$. Each neuron is driven by an intrinsic noise source $\sqrt{2(1-c)D}\xi _i(t)$ unique to each cell as well as by a *common stimulus*, $s(t) = \sqrt{2cD}\xi (t)$ which is seen by the whole population. Both the intrinsic noise sources $\xi _i(t)$ and the stimulus *s*(*t*) have spectra that are flat up to a cutoff frequency $f_{c} = 4$

2 $$\begin{aligned} S_{\xi ,\xi }(f)=S_{\xi _i,\xi _i}(f)={\left\{ \begin{array}{ll} 1, &{}\quad -f_c<f<f_c\\ 0, &{}\quad else \end{array}\right. } \end{aligned}$$

and are thus approximately white Gaussian processes, in the following also referred to as broadband noise. In Sect. 4, we use a slightly different version of the intrinsic noise, namely, an unlimited white Gaussian noise, implemented by the usual Euler–Maruyama rule (Risken 1984), but checked that the results show only small quantitative deviations when a band-limited noise is used. Parameter *c* can be used to tune how the effective intensity *D* is divided between the common stimulus and the intrinsic noise (Doiron et al. 2004; de la Rocha et al. 2007). In the case of $c=0$, the common stimulus is absent; the population neurons are driven completely by their independent intrinsic noise sources and are therefore independent in their dynamics. Conversely, when $c=1$, the input of an individual neuron consists solely of the common stimulus and, as a result, all the cells share an identical time evolution of their voltage variables. We denote the summed population output with $y(t) = \sum _{i}^N y_i(t)$.

### Postsynaptic cell (second stage)

To model the postsynaptic cell (PSC; Fig. 1, upper right corner), we again employ leaky integrate-and-fire dynamics:

3 $$\begin{aligned} \tau \dot{v} = \mu -v + \tau \sum _{k}a_k\delta (t-t_k) \end{aligned}$$

Note that the parameters of the PSC are not indicated with a subscript (in contrast to the parameters of the population neurons). The constant current bias $\mu$ is set to zero in the following unless stated otherwise. The third term on the right-hand side represents the summed output of the presynaptic population (the summation *k* runs over both neurons *i* and spike times *j* of the population model). Each spike is endowed with an individual weight $a_k$, which is either drawn from an exponential distribution with $\left\langle a_k \right\rangle = 1$ to mimic synaptic unreliability as observed in experiments, or set to the constant value $a_k = 1$ in favor of simplicity over realism. We note that an exponential function does not provide a perfect description of all experimentally observed amplitude distributions (see, e.g., Song et al. (2005), Lefort et al. (2009)) but can serve as a first-order approximation of synaptic variability and, moreover, represents a case for which analytical approximations for the driven neuron’s firing rate and spectral measures have been derived (Richardson and Swarbrick 2010) that we will use in the second part of the paper. The reset voltage is set to $v_r = 0$. The output spike train of the PSC is denoted by *x*(*t*) . The membrane time constant $\tau$ (measured in multiples of the membrane time constant $\tau _\mathrm{{POP}}$) and the threshold voltage $v_T$ are taken to be the free parameters of this model that will determine, in particular, whether the cell is responding only to highly synchronized output of the population (high threshold, short membrane time constant) and operates as a coincidence detector (CD). Alternatively, if many subsequent spikes have an accumulating effect toward firing (large membrane time constant), the PSC is referred to as an integrator cell.

### Partial synchronous output

As a proxy to the PSC acting as a CD, we consider the partial synchronous output (SO) (Kruscha and Lindner 2016; Kruscha 2017), which is a two-state process defined as a functional of the summed output of the presynaptic population:

4 $$\begin{aligned} Y_{\gamma , \varDelta }(t) = {\left\{ \begin{array}{ll} 1, &{}\quad \text {at least fraction }\gamma \text { of population} \\ &{}\quad \text {fired in }[t - \varDelta ,t] \\ 0, &{}\quad \text {otherwise} \\ \end{array}\right. } \end{aligned}$$

The parameter $\varDelta$ defines the time window in which spiking events of individual presynaptic neurons have to occur in order to be considered synchronous. Its value should be small relative to the size of the mean interspike-interval of a single presynaptic neuron ($\varDelta \ll 1 / r_0^\mathrm{{POP}}$), to decrease the probability of a single neuron firing multiple times within the window. Parameter $\gamma$, the *synchrony threshold*, specifies the minimal fraction of the population that needs to spike within $\varDelta$ in order to register a synchronous event.

The partial synchronous output distinguishes itself from a purely synchronous coding scheme in that, unlike the latter for which an event results from the simultaneous firing of the whole population ($\gamma = 1$), the demand of unanimity is relaxed ($\gamma < 1$) and an event can be triggered by a portion of the population. Although the neurons of the population share a common signal, they are also subject to a large amount of individual noise, causing their responses to vary. The margin of error provided by a lower threshold $\gamma <1$ allows the population to encode and convey information contained in the signal even if some members do not participate, as long as the number of those that do is large enough to drive the postsynaptic receiver. Even without a signal, the individual presynaptic neurons exhibit spontaneous activity and the intrinsic noise that prevents perfect synchrony can also cause occasional synchrony by chance. Therefore, in order to selectively detect only the stimulus-induced synchronous spiking, the synchrony threshold has to be set higher than the probability of a single neuron firing within the window ($\gamma > r_0^\mathrm{{POP}} \varDelta$); see also the discussion by Kruscha and Lindner (2016).

### Spectral measures

The power spectrum of the stochastic process *X*(*t*) is defined as:

5 $$\begin{aligned} S_X(f) = \lim _{T \rightarrow \infty } \frac{\left\langle \hat{X}_T(f)\hat{X}^*_T(f) \right\rangle }{T} \end{aligned}$$

where $\hat{X}_T(f)$ denotes the Fourier transform of *X*(*t*) in the time interval [0, *T*] to the frequency domain:

6 $$\begin{aligned} \hat{X}_T(f) = \int _{0}^{T}X(t)e^{-2\pi i f t}\mathrm{d}t \end{aligned}$$

and $\langle$ $\rangle$ stands for the ensemble average. The cross-spectrum of processes *X*(*t*) and *Y*(*t*) is defined as:

7 $$\begin{aligned} S_{X,Y}(f) = \lim _{T \rightarrow \infty } \frac{\left\langle \hat{X}_T(f)\hat{Y}^*_T(f) \right\rangle }{T} \end{aligned}$$

If *X*(*t*) is the output of a system with known transfer function *K*(*t*), the Fourier transform of which defines the *susceptibility*, $\chi (f) = \mathcal {F}\{K(t)\}$, the cross-spectrum can be expressed in terms of the power spectrum of the system’s input $S_Y(f)$ as follows:

8 $$\begin{aligned} S_{X,Y}(f) = \chi (f) S_Y(f). \end{aligned}$$

The coherence function between processes *X*(*t*) and *Y*(*t*) is a linear measure of information transmission

9 $$\begin{aligned} C_{X,Y}(f) = \frac{|S_{X,Y}(f)|^2}{S_X(f) S_Y(f)}. \end{aligned}$$

It attains values between 0 and 1 and can be regarded as a (squared) correlation coefficient (covariance over product of standard deviations) in the frequency domain. For Gaussian stimuli in a broad frequency band $[0,f_c]$, as used in this paper, the coherence also provides a lower bound to the mutual information rate, given by $R_{\text {info}}=-\int _0^{f_c} \mathrm{d}f \log _2(1-C(f))$ (Rieke et al. 1993; Gabbiani 1996) [an improved lower bound formula for slow stimuli is derived and discussed by Voronenko and Lindner (2018)]. A high coherence value in a certain frequency band indicates a strong information transmission of the corresponding stimulus component.

In order to quantitatively distinguish between low-pass and band-pass/high-pass information filtering, the *quality* of information filtering (Kruscha 2017)

10 $$\begin{aligned} Q = 1 - \frac{C(0)}{C(f_\mathrm{{max}})} \end{aligned}$$

can be applied. The coherence function of a system which acts as a low-pass filter has a maximum at zero frequency ($C(f_\mathrm{{max}}) \approx C(0)$), resulting in a *Q* value close to or equal to zero. In contrast, band-pass information filters are characterized by a pronounced peak at some finite frequency. A large difference between the maximum and the amplitude at $f=0$ leads to values of *Q* close to one. Therefore, for the SO to act as a good proxy for the CD, its information filtering quality should be near 1, matching that of the high-pass operational regime of a typical CD.

## Tailoring partial synchrony to mimic coincidence detector information transmission

The PSC as defined in Eq. (3) generates spikes indirectly in response to the broadband stimulation of the first-stage population. If we do not change the parameters of the stimulus (amplitude, bandwidth) and of the population (time constant, noise intensity, internal bias), the coherence of the PSC’s spike train and the input stimulus will depend only on the membrane time constant and threshold value. Can we mimic the coherence function for different values of the parameters $v_T$ and $\tau$ with that of the partial synchronous output if we adapt the synchronization time window $\varDelta$ and the synchrony threshold $\gamma$ accordingly?

**Fig. 2:**
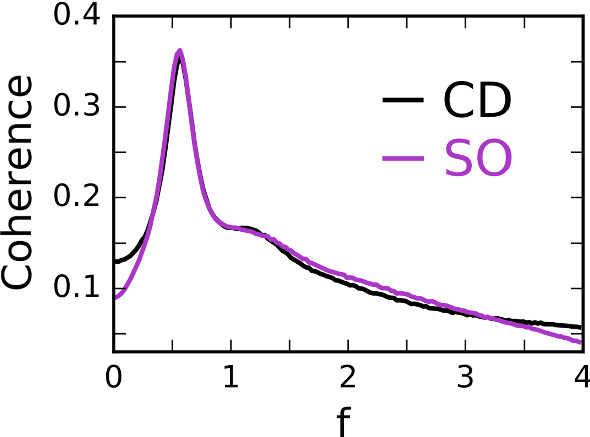
Coherence function comparison. The coherence function of a PSC acting as a CD (black) with a short membrane time constant ($\tau =0.1$) and high voltage threshold ($v_T=10$). The optimally matched SO coherence function (purple) is likewise dependent on a time parameter ($\varDelta _\mathrm{{opt}}=0.18$) and a threshold parameter ($\gamma _\mathrm{{opt}}=0.19$). Although the match is not perfect, there is strong agreement in the region of interest around the peak (color figure online)

In Fig. 2, we show for one example of a PSC in CD mode that it is possible to achieve a strong similarity of the coherence functions of the PSC and the SO in a frequency band around the maximal coherence (Bostner 2019). In the following, we demonstrate that this is not an isolated case, but that generally, given a postsynaptic detector with certain values of $v_T$ and $\tau$, corresponding synchrony parameters, $\varDelta$ and $\gamma$ can be found which minimize the difference between the two coherence functions in a certain frequency window.

### Synchrony parameter search

**Fig. 3:**
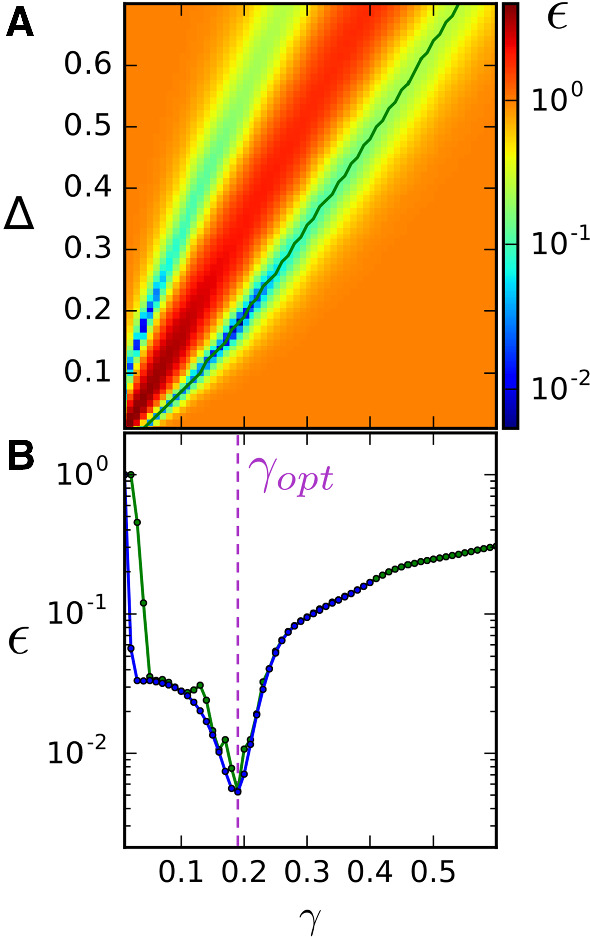
Demonstration of the search process. **a** Relative squared deviation $\epsilon$ over the parameter space ($\gamma , \varDelta$) of the SO with sampling resolution $d\gamma = 0.01$ and $d\varDelta = 0.01$ for fixed parameters of the coincidence detector: $\tau =0.1$, $v_T=10$. Dark shades of blue indicate the position and shape of the two minima with a pronounced maximum between (dark red). We focus here on the minimum to the right of the maximum, corresponding to a parameter regime where the SO is tuned to detect synchronous spiking. The minimum to the left of the maximum, at which the SO detects common silence, is neglected. The green line indicates the position of the minimum as a function of $\gamma$, $\epsilon (\gamma , \varDelta _\mathrm{{opt}}(\gamma ))$. **b** Minimum of $\epsilon$ as a function of $\gamma$ for initial (green: $d\varDelta =0.01$) and increased (blue: $d\varDelta =0.0005$) sampling resolutions. Although in both cases the same global minimum is found (purple dashed line), the descent to the minimum is smoother with a smaller sampling step and, therefore, the values for $\gamma _\mathrm{{opt}}$ and $\varDelta _\mathrm{{opt}}$ can be more reliably determined (color figure online)

The similarity of the coherence functions of the postsynaptic cell $C_\mathrm{{CD}}$ and the partial synchronous output $C_\mathrm{{SO}}$, can be quantified by a relative squared deviation

11 $$\begin{aligned} \epsilon (C_\mathrm{{CD}}, C_\mathrm{{SO}}) = \frac{\int _0^{f_\text {sim}}(C_\mathrm{{CD}}(f) - C_\mathrm{{SO}}(f))^2 \mathrm{d}f}{\int _0^{f_\text {sim}} C^2_\mathrm{{CD}}(f)\mathrm{d}f}. \end{aligned}$$

$f_\text {sim}$ characterizes the size of the interval on which the coherence functions are compared; in the following we will use $f_\text {sim}=f_c=4$. Smaller values of $\epsilon$ indicate greater similarity between the coherence functions.

In order to compare the information-filtering properties of both models, the PSC parameter values are held fixed and a grid search is performed over the parameter space ($\gamma , \varDelta$) of the SO, computing the squared deviation $\epsilon$ at each point from the coherence curves obtained by numerical simulations. Figure 3a shows the resulting $\epsilon$ values across the parameter space for the fixed PSC values $\tau =0.1$ and $v_T=10$ used throughout this subsection. The optimal values of the SO parameters, $(\gamma _\mathrm{{opt}}, \varDelta _\mathrm{{opt}})$, are the coordinates of the global minimum of $\epsilon$. As can be seen in the figure, there are actually two local minima corresponding to parameter values which cause synchrony detection and values which cause common silence detection (for a discussion of this symmetry, see also Kruscha and Lindner (2016)). The two minima are separated by a maximum, where the focus shifts from common silence to common firing. In the following, we will only consider the part of the parameter space which corresponds to common firing, or synchrony in the classical sense, which is shown mostly in the lower right triangle of Fig. 3a.

In order to get a better understanding of the landscape around the minimum, the dimensionality can be reduced by plotting the minimum of $\epsilon$ as a function of $\gamma$ only; $\varDelta$ is determined from the corresponding minimum value of $\epsilon$ for a given $\gamma$, shown by the green line running almost along the diagonal in Fig. 3a. The minimum $\epsilon$ values of that line are shown in Fig. 3b at two sampling resolutions for delta, $d\varDelta =0.01$ in green and $d\varDelta =0.0005$ in blue. The resolution of gamma, which is limited by the size of the presynaptic population, is in both cases $d\gamma = 0.01$. A gradual increase in the resolution with which possible candidates for optimal matches is sampled in the $(\gamma , \varDelta )$ parameter space leads to a suppression of numerical artifacts and a better localization of the minimum, and therefore optimal $\gamma$, which is indicated by the dashed line in Fig. 3b. Once the optimal SO parameters are found, the coherence functions of the CD and SO can be plotted for comparison.

As can be seen in Fig. 2, the minimization of the relative squared deviation leads to a particularly close match of the two coherence functions in the frequency interval around the peak, whereas at smaller and larger frequencies deviations are more apparent.

### Relations between parameters of detector (PSC) and proxy (SO)

Using the above process of finding optimal SO parameter matches for given PSC parameters, the relationships among the parameters of the two types of output can be found by systematically varying one of the PSC parameters while holding the other fixed.

**Fig. 4:**
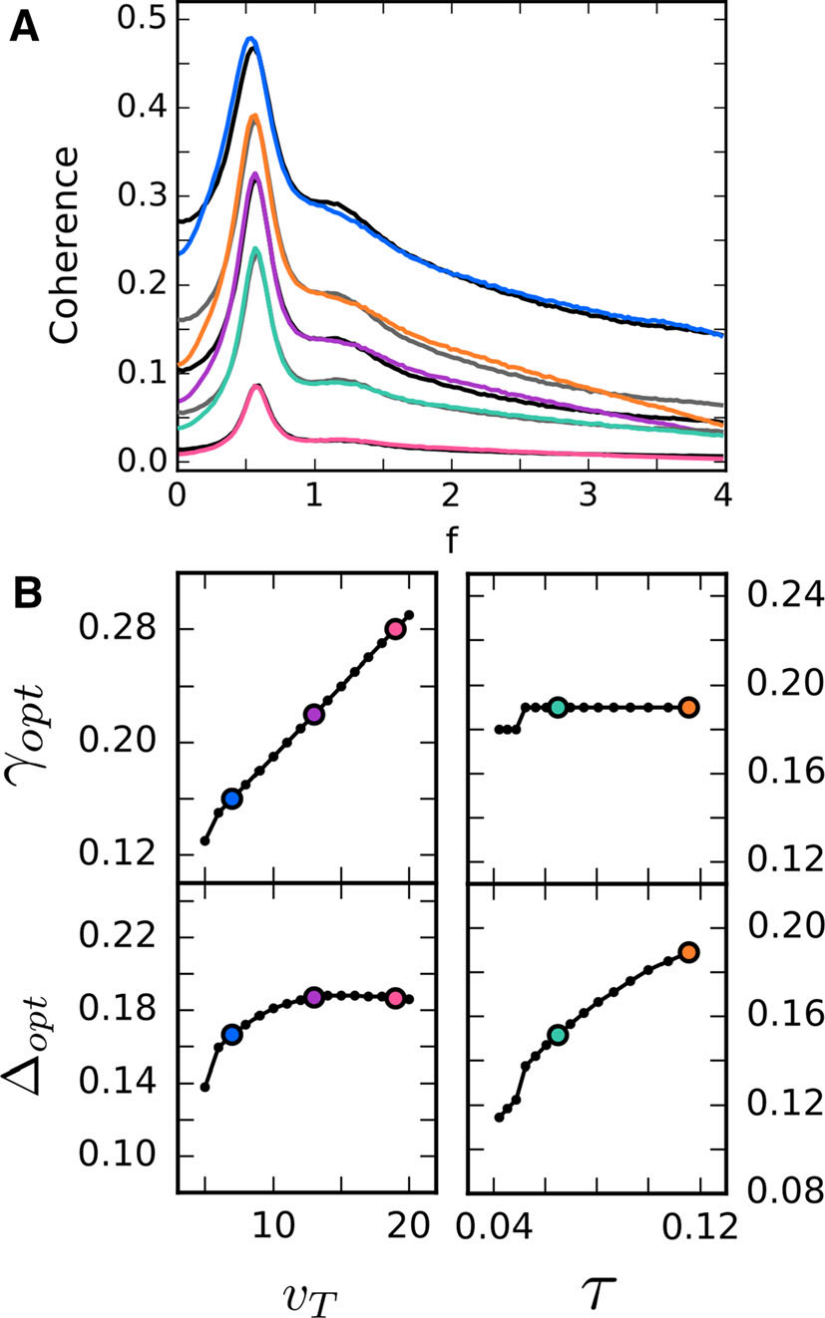
Extracted dependencies of $\gamma _\mathrm{{opt}}$ and $\varDelta _\mathrm{{opt}}$ on $\tau$ and $v_T$. **a** Selected examples of the comparison of the coherence functions of the CD (black: $v_T$ varied, $\tau = 0.1$; gray: $\tau$ varied, $v_T= 10$) and the optimally matched SO (blue, purple, pink: $v_T$ varied; seafoam green, orange: $\tau$ varied). **b** Top: $\gamma _\mathrm{{opt}}$ increases somewhat linearly with $v_T$ (left), whereas it is mostly independent of the chosen value of $\tau$ (right), confirming the connection between the threshold parameters. Bottom: The value of $\varDelta _\mathrm{{opt}}$ is largely unaffected by changes in $v_T$ and instead increases roughly linearly with its time-parameter counterpart $\tau$ (color figure online)

Figure 4a shows some examples from the variation of both $v_T$ and $\tau$ for illustration purposes. The black lines are the PSC coherences with constant $\tau$ for different values of $v_T$ and the blue, purple, and pink lines are the SO coherences, which are all close to the corresponding black line. The gray lines show the PSC coherences for fixed $v_T$ and two different values of $\tau$, along with the matched SO coherences in green and orange.

The optimal SO parameters of all variations can then be plotted against the PSC parameters as shown in Fig. 4b, where the highlighted points represent the matched parameters of the selected examples in Fig. 4a. An approximately linear relation between temporal ($\tau$ and $\varDelta$: bottom right) as well as between threshold ($v_T$ and $\gamma$: top left) parameters becomes apparent. In contrast, there is only a weak dependence between the threshold and temporal parameters (bottom left and top right).

**Fig. 5:**
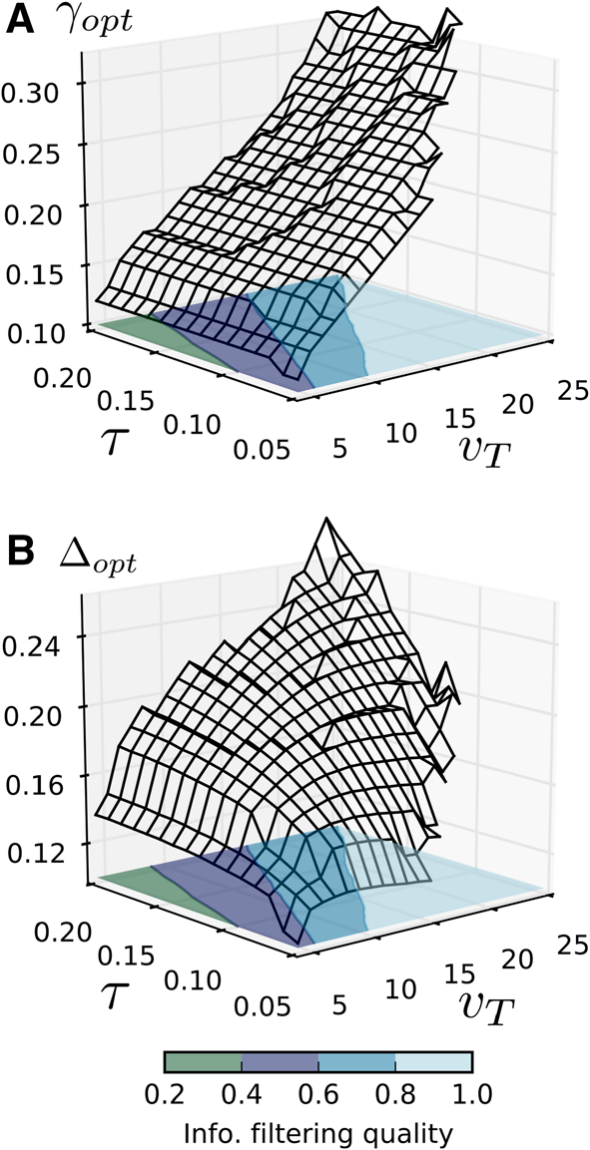
Dependence of $\gamma _\mathrm{{opt}}$ and $\varDelta _\mathrm{{opt}}$ on $\tau$ and $v_T$. The 3D plots show the results of a grid search for optimal SO matches in a region of the ($v_T, \tau$) PSC parameter space. The colored contour plots indicate the quality of information filtering *Q* of the PSC, independent of the SO values and is therefore the same in both A and B. Of particular interest are both blue regions, which have large Q values and indicate band-pass filtering. a The dependence of $\gamma _\mathrm{{opt}}$ is qualitatively similar to Fig. 4 and holds even for low information quality regions, suggesting a robust and exclusive connection between the threshold parameters. **b** The dependence of $\varDelta _\mathrm{{opt}}$ is also as previously shown, but only for regions with large quality of information filtering. The results for the small area around ($v_T = 25, \tau = 0.05$) were excluded because the variances in the estimations became as large as the amplitudes due to the finite size of the ensembles ($10^4$ realizations) used in the numerical simulations

The search for optimal matches can be extended to a grid search in a region of the PSC parameter space, ($v_T, \tau$), as shown in Fig. 5. For every sampled point in that space, one of the optimal SO parameters is found (surface plot) as well as the value of *Q*, the quality of information filtering (color-coded contour plot). *Q* provides a means of distinguishing regions in parameter space where the postsynaptic cell acts primarily as a band-pass information filter from those where it performs low-pass filtering. When restricted to the band-pass (blue) regions, the grid search results corroborate the results of Fig. 4: a roughly linear dependence between analogous parameters of both models and little-to-no dependence between dissimilar parameters.

In light of the results of the next section (especially, Sect. 4.2), one may wonder how strongly the mapping procedure depends on the randomization of the synaptic amplitudes $a_k$ that we have exclusively used so far. In appendix, Sect. 1, we show equivalent results for the case of constant amplitudes when matching the SO and CD coherence functions ($a_k\equiv 1$) and for the relation between time-window and threshold parameters. The results illustrate that the mapping does not qualitatively hinge on having stochastic amplitudes, but that the exact choice of amplitude distribution quantitatively changes the relation between synchrony and CD parameters, as can be somewhat expected.

## Approximating coincidence detector coherence explicitly

### Linear response to shot-noise input from population

In the previous section, the focus was on finding the best parameters for the SO, such that it acts as a good proxy for the postsynaptic coincidence detector cell. In this section, we return to the PSC itself and develop an alternative to the proxy description: a direct analytical approximation of the coherence between the PSC’s output spike train and the stimulus agitating the first-stage population (Bostner 2019).

For either the population or the PSC, it is assumed that the respective activity (population or firing rate) can be well-approximated by the *linear-response ansatz*:

12 $$\begin{aligned} \left\langle z(t) \right\rangle\approx & {} \left\langle z(t) \right\rangle _{s(t) \equiv 0} + \int _{-\infty }^{t} K(t - t')s(t') ~\mathrm{d}t' \nonumber \\ \left\langle \hat{z}(f) \right\rangle\approx & {} \chi (f) \hat{s}(f),\quad \forall f>0 \end{aligned}$$

In other words, the stationary rate (with $s(t)=0$ or equivalently $c=0$) plus the convolution of the signal with a first-order causal filter *K*(*t*), whose Fourier transform is the susceptibility $\chi (f)$. For the second-order statistics, i.e., the power spectrum, we use another common ansatz:

13 $$\begin{aligned} S_{z} \approx S_{z,0} + |\chi |^2 S_{s} \end{aligned}$$

where $$S_{z,0}$$ is the unperturbed power spectrum and $$ S_{s}$$ is the stimulus power spectrum; for a discussion of the validity of this approximation, see Lindner et al. (2005a).

All neurons in the system are LIF neurons for which analytical solutions for the spectra exist when they are driven by Gaussian white noise (Lindner and Schimansky-Geier 2001; Lindner et al. 2002; Brunel et al. 2001), including the power spectrum $S_{\text {GN}}$ and susceptibility $\chi _{\text {GN}}$ (for the corresponding expressions, see appendix, Sect. 2). In the case of the population, the neurons are subject to Gaussian white noise and hence these results can be applied. For the PSC, the incoming spike trains of the population are closer to what is known as Poissonian *shot noise*, for which there again exist solutions for the power spectrum $S_{SN}$ and susceptibility $\chi _{\text {SN}}$ (Richardson and Swarbrick 2010; Droste 2015; Droste and Lindner 2017a) that we will use below.

In order to calculate the coherence of the PSC with the signal using Eq. (9), their power spectra and cross-spectrum are needed. The power spectrum of the common white-noise signal is proportional to its intensity, $S_{s}(f) = 2Dc$. Using Eq. (8), the cross-spectrum between the common stimulus, *s*(*t*), and the output spike train of the PSC, *x*(*t*), can be approximated by combining the linear responses of the population (Gaussian ansatz, $\chi _{\text {GN}}$) and the PSC (shot-noise ansatz, $\chi _{\text {SN}}$). The common stimulus modulates the population rate, which in turn is seen by the PSC as rate-modulated shot noise, resulting in the cross-spectrum:

14 $$\begin{aligned} S_{xs} = \chi _{\text {PSC}}\chi _{\text {POP}}S_{s} \approx 2DcN \chi _{\text {SN}} \chi _{\text {GN}} \end{aligned}$$

here, $\chi _{\text {POP}}(f) = N\chi _{\text {GN}}(f)$ is the susceptibility of the presynaptic population to the common stimulus evaluated using the intensity *D* of the total noise including the signal (see Lindner et al. (2005b)). This susceptibility is proportional to the single-neuron susceptibility because all *N* neurons are uncoupled. The function $\chi _{\mathrm{PSC}}(f) \approx \chi _{\text {SN}}(f)$ is the susceptibility of the PSC to the modulation of the firing rate of its input spike train approximated by the shot-noise susceptibility.

The power spectrum of the PSC can be found using a linear response approximation for the two stages of transmission. Beginning with the population and applying Eq. (13), the power spectrum of its output *y*(*t*) is estimated by:

15 $$\begin{aligned} S_{y}= & {} \left\langle \hat{y} \hat{y}^{*} \right\rangle = \sum _i \sum _j \left\langle \hat{y}_i \hat{y}_j^* \right\rangle \nonumber \\= & {} N S_{y_1} + N (N - 1) S_{y_1, y_2}\nonumber \\\approx & {} N (S_{y_1, 0} + |\chi _{\text {GN}}|^2 S_s) + N (N - 1) \left\langle \chi _{\text {GN}}\hat{s}\chi _{\text {GN}}^*\hat{s}^* \right\rangle \nonumber \\= & {} N S_{\text {GN}} + N^2|\chi _{\text {GN}}|^2 S_s \end{aligned}$$

(Cross-correlation terms are only due to the common stimulus).

In the next processing stage, the output of the population is the spike train input to the PSC. The analytical solution for the power spectrum of an LIF neuron receiving shot noise input introduced earlier, $S_{\text {SN}}$, requires the input to have homogeneous Poisson statistics and thereby a flat power spectrum. However, Eq. (15) is not generally a flat spectrum, and therefore what the PSC sees is not the kind of noise for which we know the susceptibility and power spectrum. Nevertheless, as mentioned above we will use the shot-noise susceptibility as an approximation for the PSC’s susceptibility to the population rate modulation. This still leaves the problem of how to approximate the PSC’s power spectrum if the input spike trains do not have Poissonian statistics.

In order to get an approximation for the power spectrum, we use a different kind of linear response ansatz in the Fourier domain:

16 $$\begin{aligned} S_{x}\approx & {} S_{\text {SN}} + |\chi _{\text {SN}}|^2(S_{y} - S_{\text {hP}})\nonumber \\\approx & {} S_{\text {SN}} + N|\chi _{\text {SN}}|^2(S_{\text {GN}} + N|\chi _{\text {GN}}|^2 S_{s} - r_{\text {GN}}) \end{aligned}$$

where $r_{\text {GN}}=r_0^\mathrm{{POP}}$ is the mean firing rate of an LIF neuron driven by Gaussian white noise, and therefore of a single population neuron, and $S_{\text {hP}}=N r_0^\mathrm{{POP}}$ is the power spectrum of a (hypothetical) Poissonian spike train that has the same overall firing rate as the population output. In Eq. (16), the difference between the true input spectrum and the Poissonian spectrum is treated as a small perturbation that is corrected with a response term given by the rate-modulation susceptibility (Schwalger 2019); for a detailed inspection of when this approach works and how it can be (approximately) derived in the low-frequency limit, see Bostner (2019).

Plugging in the results for the spectra derived above, the coherence of the PSC is approximated as follows:

17 $$\begin{aligned} C_{x, s} \approx \frac{N^2 |\chi _{\text {SN}} \chi _{\text {GN}}|^2 S_s}{S_{\text {SN}} + N |\chi _{\text {SN}}|^2 ( S_{\text {GN}} + N|\chi _{\text {GN}}|^2 S_{s} - r_{\text {GN}})} \end{aligned}$$

Note that the power spectrum $S_{\text {SN}}$ and the susceptibility $\chi _{\text {SN}}$ also depend on the size of the population *N*, but their dependence is more indirect and cannot be expressed by a simple prefactor.

**Fig. 6:**
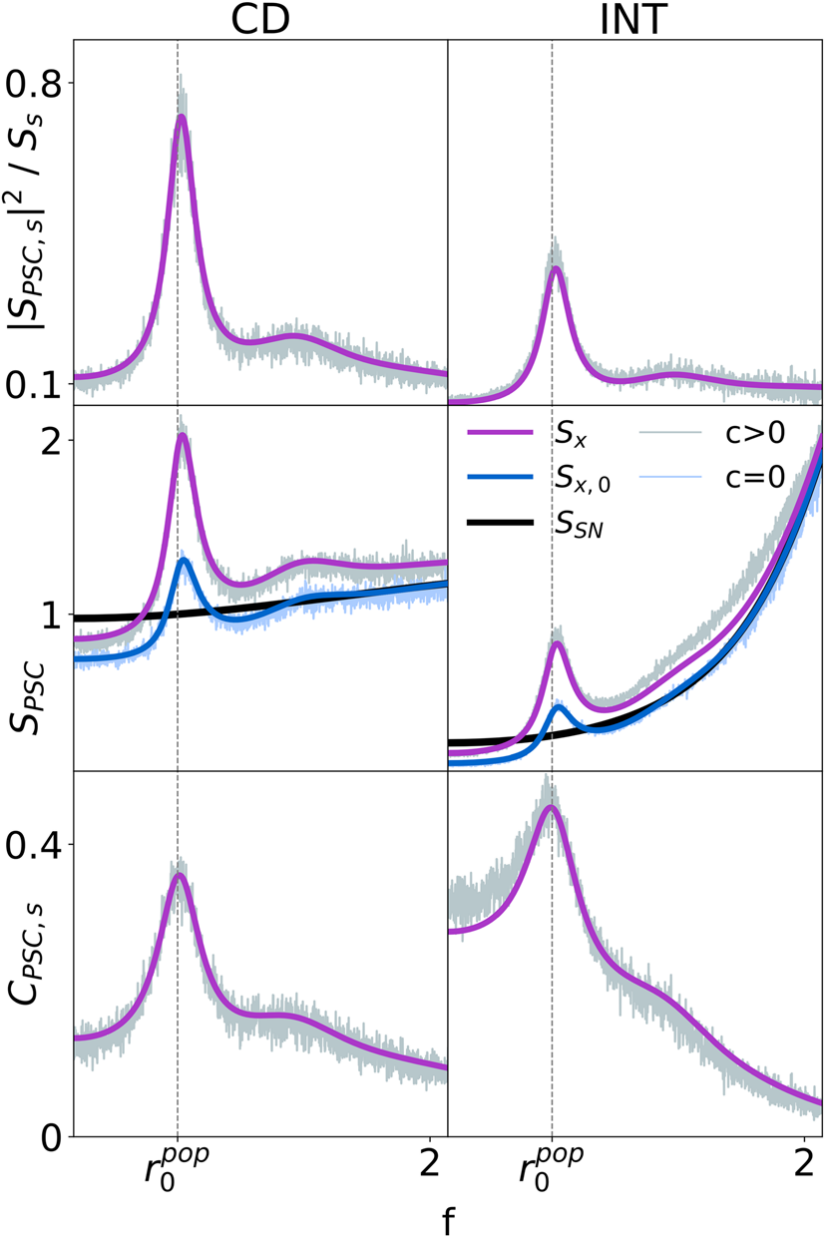
Approximations for the power and cross-spectra and the coherence function compared to numerical simulations. Left: fluctuation-driven regime, $\tau =0.1$, $v_T=10$. Right: mean-driven regime, $\tau =10$, $v_T=20$. Top: Square of cross-spectra amplitudes normalized by the power spectrum of the common stimulus. Middle: Power spectra with the common stimulus [gray: simulation; magenta: theory] and without [light blue: simulation; blue: theory]. For comparison, the analytical expression for the power spectrum of the PSC driven purely by Poisson shot-noise ($S_{\text {SN}}$) is shown in black. Bottom: Coherence functions. 1000 simulations, $T=1000$, $\mathrm{d}t=10^{-3}$, $\left\langle a_k \right\rangle =1$ (color figure online)

The results for the cross-spectrum, Eq. (14), power spectrum, Eq. (16), and coherence, Eq. (17), of the PSC in two different modes are shown in Fig. 6. In the left column, the PSC acts as the coincidence detector encountered above. As a CD, the neuron engenders the characteristic behaviors of quick memory loss and being driven by fluctuations of the input ($\left\langle a_k \right\rangle N r_0^\mathrm{{POP}} \tau < v_T$), i.e. the mean input from the population alone does not drive the PSC over the threshold. The input fluctuations in this case represent synchronized behavior of the population. These properties are realized by a short time constant ($\tau = 0.1$) and a threshold which is high enough to discount small, chance synchronicity but low enough to capture desired concurrence ($v_T = 10$).

In contrast, when the PSC acts as an integrator (INT) as shown in the right column of Fig. 6, it retains information about past events over longer periods of time due to a larger time constant ($\tau =10$). Instead of the fluctuation-driven regime, the INT operates in the mean-driven regime ($\left\langle a_k \right\rangle N r_0^\mathrm{{POP}} \tau > v_T$), in which it is continually reacting to the population activity as a whole, instead of singling out coincident events. Note that with the chosen high threshold for the INT ($v_T=20$), the mean firing rates of INT and CD are not very different ($r_\mathrm{{INT}} = 2.7, r_\mathrm{{CD}}=1.5$).

The top row of Fig. 6 shows the cross-spectra of the PSC with the stimulus, normalized by the stimulus’ power spectrum. The cross-spectrum is a measure of the correlation between the output of the PSC and the common stimulus and has a similar shape for both the CD and INT. It exhibits a peak at the individual mean rate of the population neurons as expected and is accurately described by Eq. (14).

The power spectra (middle row, Fig. 6) for the cases of the signal being present ($c>0$: theory in magenta, simulations in gray) or absent ($c=0$: theory in blue, simulations in light blue) are shown and compared to the LIF spectrum for homogeneous shot noise (black). When there is no common stimulus ($c=0$), the difference between the blue and black curves is solely due to deviations of the population’s activity from Poisson statistics. Most markedly, the peak in the population’s activity spectrum (see below: Fig. 8, middle) results in a similar peak in the PSC’s power spectrum at $r_0^\mathrm{{POP}}$. The regularity of population firing is also reflected in the depressed spectrum at frequencies lower than $r_0^\mathrm{{POP}}$, which is smaller than that of the shot-noise spectrum. For this reason, a subtractive term is needed in the theory and is achieved by the correction term in Eq. (16). The population’s spectrum at higher frequencies, on the other hand, converges to the firing rate (as for a Poisson process), and therefore the PSC’s spectrum for $c=0$ approaches the shot-noise theory in that limit. When applying the common stimulus ($c>0$), it is first filtered by the population and results in an upward shift in the PSC’s spectrum, especially around $f \approx r_0^\mathrm{{POP}}$. Our theoretical ansatz, Eq. (16), describes the PSC’s spectrum in all cases reasonably well.

Finally, dividing the cross-spectra (top) by the power spectra (middle) yields the coherence functions (bottom), displaying again good agreement between simulations and theory. The CD encodes little information except in a narrow frequency band around the population’s individual rate. Such band-pass information filtering is characteristic of a neuron tuned to detect synchrony and comes at the expense of the overall information transmission. In contrast, in the INT mode the PSC preferentially conveys low-frequency stimulus information. However, the expected peak at $f \rightarrow 0$ and accompanying monotonic decrease, the hallmarks of a low-pass filter, are missing. Instead, there is still a peak at $r_0^\mathrm{{POP}}$ (although not as pronounced) and thus the INT can be regarded as a (imperfect) band-pass information filter. The reason for this behavior in the INT mode is explained in the following section. Before we come to this, we study the effect of an additional bias current on spectral measures and the coherence of the PSC.

**Fig. 7:**
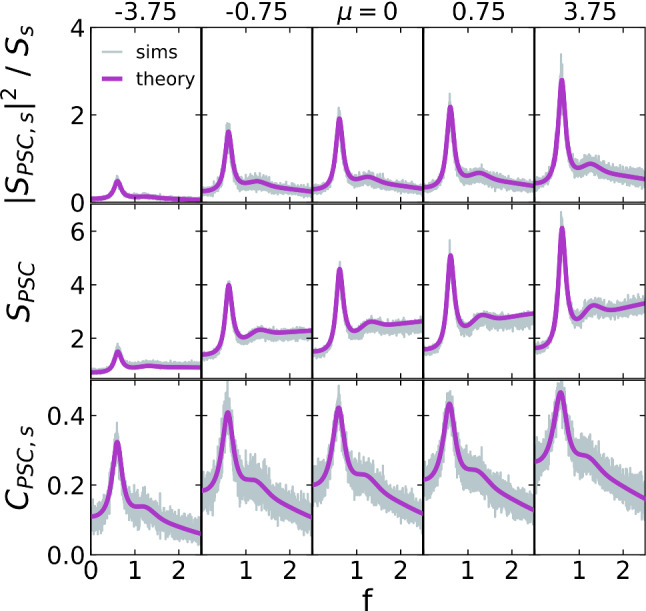
Spectra and coherence functions of the PSC in CD mode in response to different biases, $\mu$. The external input is swept from negative to positive values (left-to-right) and all other parameters are left unchanged. Top: Squared magnitude of the cross-spectrum of PSC and signal, normalized by the stimulus’ power spectrum. Middle: PSC power spectrum. Bottom: Coherence between PSC output and the common stimulus. 500 simulations, $T=1000$, $\mathrm{d}t=10^{-3}$, $v_T=7.5$, $\tau =0.1$, $\left\langle a_k \right\rangle =1$

So far, we used a value of $\mu =0$ for the external current. It is not clear *a priori*, however, whether the PSC operates in a fluctuation- or mean-driven regime; this can be controlled in our model, Eq. (3), by the parameter $\mu$. We therefore test our theory for cross-spectra (top row), power spectra (middle row), and coherence functions (bottom row) for different values of $\mu$ in Fig. 7, starting with a value of $\mu =-3.75$ which puts the PSC deep into the fluctuation-dominated regime ($\left\langle a_k \right\rangle N r_0^\mathrm{{POP}} \tau + \mu < v_T$ with $v_T=7.5, r_0^\mathrm{{POP}}\approx 0.6, \left\langle a_k \right\rangle =1, \tau =0.1$) and ending with $\mu =3.75$, a PSC in the mean-driven regime, for which $\left\langle a_k \right\rangle N r_0^\mathrm{{POP}} \tau + \mu > v_T$. Both cross- and power spectra increase with growing bias. As a result, the coherence maintains a peak but i) this peak becomes less pronounced and ii) the amount of total information increases. Regarding the first observation, we note that the ratio of the peak’s magnitude to the average coherence decreases (around 3:1 on the left and less than 2:1 on the right), indicating that the PSC loses some of its effectiveness as an information filter. For all bias values shown, the theory tracks the simulation results rather well.

### Effect of synaptic weights on information transmission

The lack of a low-pass profile in the INT coherence of Fig. 6 is somewhat surprising, given that the parameters of the PSC in this case should result in an integrator, which should relay low-frequency information with high fidelity. In the following, we demonstrate that the weak band-pass information filtering is an effect of endowing the output spikes of the population with random amplitudes. To this end, we analyze the coherence function of the latter with the broadband stimulus and compare it to the coherence of the total population output with constant spike amplitudes (referred to as *all-spikes* coherence by Middleton et al. (2009), Sharafi et al. (2013), Grewe et al. (2017)). These coherence functions are upper bounds for the coherence functions of the PSC driven by the population spikes with random and constant amplitudes, respectively.

**Fig. 8:**
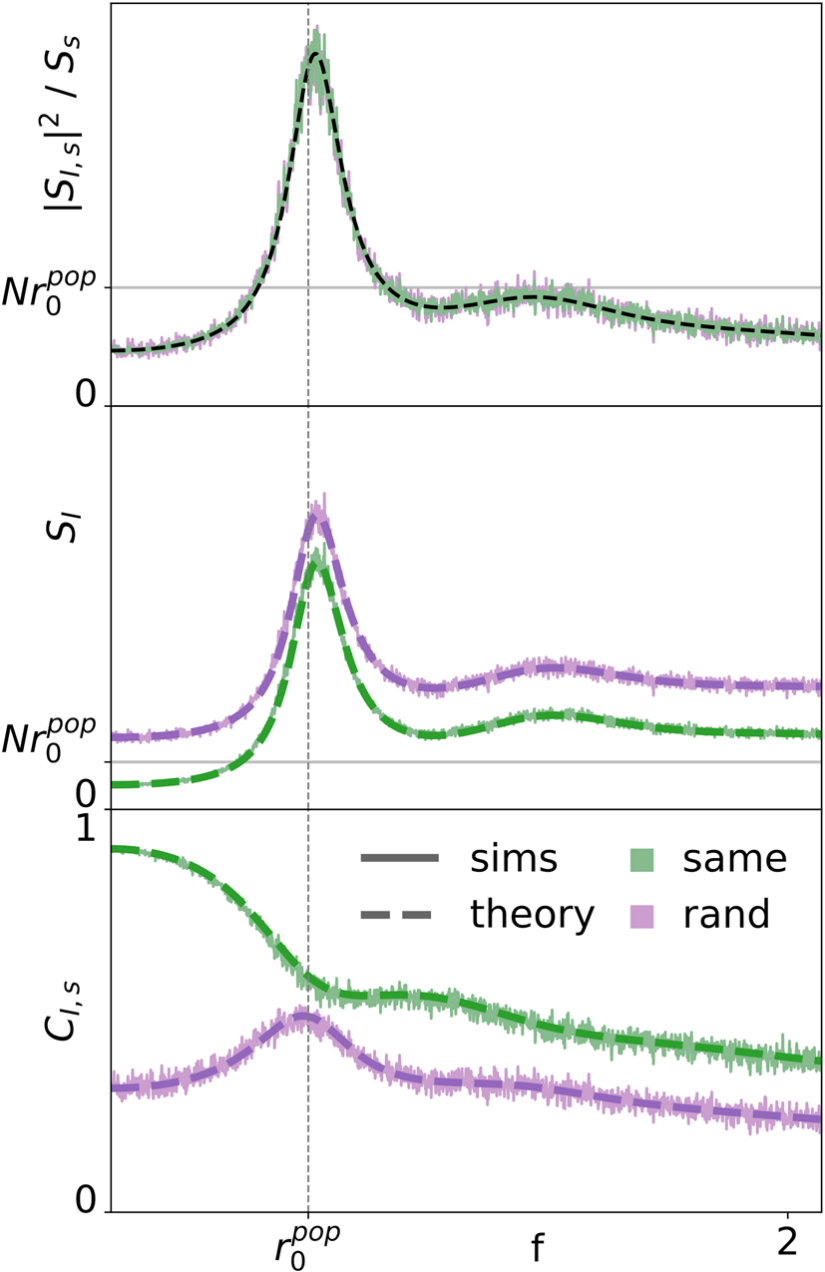
Spectra of weighted PSC input, I(t). [green: all weights are the *same*; purple: each spike receives a random (*rand*) weight] Top: The cross-spectrum is unaffected by the weight scheme and therefore the same for both (theory from Eq. (19): black, dashed line). Middle: The power spectrum (theory found in Eq. (20): dashed lines) for random weights is shifted upward across frequencies by an additive white noise. Bottom: The *same* coherence (theory from Eq. (21): dashed line) retains most low-frequency information due to low power in that region. The *rand* coherence shows significant low-frequency information loss due to the comparatively high power at low frequencies. The additive white noise drowns out information in all frequency bands except around $r_0^\mathrm{{POP}}$ (color figure online)

In the following, the coherence of the input current

18 $$\begin{aligned} I(t) = \sum _{k} a_k \delta (t - t_k) \end{aligned}$$

is compared for the cases that $a_k\equiv 1$ (constant amplitude case) and $a_k$ coming from an exponential distribution with $\left\langle a_k \right\rangle =1$.

A comparison of the spectra and coherence functions of the weight schemes are shown in Fig. 8. The cross-spectrum (normalized by the signal power; top) and power spectrum (middle) for both schemes exhibit a peak at the single-neuron mean rate, $r_0^\mathrm{{POP}}$. However, the coherence (bottom) displays the peak for random weights only. As calculated in appendix, Sect. 3, the cross-spectrum

19 $$\begin{aligned} S_{Is} = \left\langle a_k \right\rangle N \chi _{\text {GN}} S_s \end{aligned}$$

is the same for both cases (see top panel), and therefore clarification is sought in the power spectrum. The latter can be approximated (see also appendix, Sect. 3) as

20 $$\begin{aligned} S_{I} = N\left\langle a_k \right\rangle ^2\left[ (N-1)|\chi _{\text {GN}}|^2 S_{s} + S_{\text {GN}}(f) + r_0^\mathrm{{POP}} C_{V,a}^2\right] \end{aligned}$$

The difference between the two cases is in the coefficient of variation of the amplitudes, which is $$ C_{V,a}^2=1$$ for the exponential distribution but $$ C_{V,a}^2=0$$ if the amplitudes are all the same (constant).

As a consequence of the additional offset in the power spectrum in the case of random weights, the coherence function obtained from the spectral measures above,

21 $$\begin{aligned} C_{I,s} = \Bigg [ \frac{N - 1}{N} + \frac{S_{\text {GN}}+r_0^\mathrm{{POP}}C_{V,a}^2}{N |\chi _{\text {GN}}|^2 S_{s}} \Bigg ]^{-1} , \end{aligned}$$

displays a peak at the frequency where the cross-spectrum is maximized, $r_0^\mathrm{{POP}}$. This is a consequence solely of the flattening of the spectrum by the randomization of the synaptic amplitudes; no coincidence detection is involved. This effect is confirmed by numerical simulations in Fig. 8 and demonstrates preferential encoding of a narrow frequency band already at the input side of the PSC with random amplitudes.

**Fig. 9:**
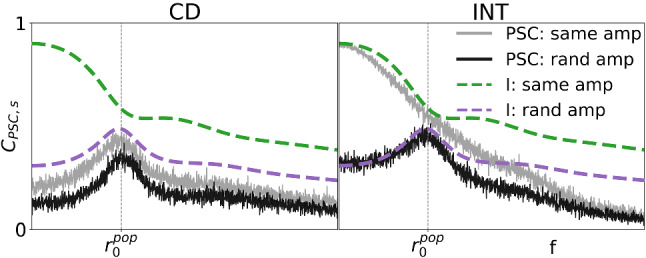
PSC coherence with constant versus random synaptic weights. [Random amplitudes: Simulations of PSC (black) versus the input theory (Eq. (21), purple). Same amplitude: Simulations of PSC (gray) versus the input theory (Eq. (21), green).] Left: In coincidence-detector (CD) mode, the desired band-pass peak is achieved with constant or random weights. Right: In integrator (INT) mode, the type of synaptic weight makes a marked difference. In both weight schemes, the PSC transmits most available low-frequency information and attenuates at higher frequencies. If all incoming spikes have the same weight, the INT is a prototypical low-pass filter. In contrast, the INT receiving random weights has poor fidelity at low frequencies and a peak at a finite frequency, $r_0^\mathrm{{POP}}$. Parameters are the same as those in Fig. 6 (color figure online)

As mentioned above, the derived coherence functions of the input constitute upper bounds for the corresponding coherence functions of the PSC with the stimulus. In Fig. 9, coherence functions calculated from simulations of the PSC for both weight schemes in the CD (left) and integrator (right) modes are compared to the calculated upper bounds from Eq. (21). A peak in the coherence is always observed with random amplitudes (purple and black curves), irrespective of the parameters of the PSC. With constant-amplitude population input, the PSC shows a low-pass coherence in the integrator mode (right, gray curve) but a narrow-band coherence in CD mode (left, gray curve). Directly comparing the PSC’s performance with the incoming information upper bounds gives a clear idea how PSC parameters generate (for input with a constant amplitude) and sharpen (for input with random amplitudes) the information filtering effect.

## Summary and conclusions

We achieved two goals in this paper. First of all, by extensive numerical simulations of a simple two-stage neural system, we identified the relation between the information linearly encoded by the coincidence detector at the second stage to that encoded by the synchronous output of the first stage. As conjectured in the literature, we can take the latter as a proxy for the former if the parameters of the partial synchronous output are appropriately chosen. Specifically, we found that the time window $\varDelta$ and threshold $\gamma$ of our synchrony definition scale approximately linearly with the time constant $\tau$ and voltage firing threshold $v_T$ of the PSC over a physiologically relevant range, matching our intuition about the meaning of $\varDelta$ and $\gamma$ (Kruscha and Lindner 2016). Conversely, we can also regard the PSC as a suitable synchrony detector—adapting the PSC parameters, we could tune the output to optimally pick up synchronous spikes according to a given synchrony parameter pair $\varDelta ,\gamma$. This is what presumably happens in the electrosensory system of weakly electric fish: sensory receptor cells (P-units) target neurons in multiple maps in the electrosensory lateral line lobe; target cells vary in their cellular properties across the maps and thus encode information in the frequency bands of a broadband stimulus differently, which can be traced back to their distinct responses to synchronous input (Grewe et al. 2017).

The second goal was to develop an alternative approximation for the coherence function of the PSC which does not rely on the proxy approximation of Sharafi et al. (2013), Kruscha and Lindner (2016). To this end, we applied linear response theory combined with a novel approach to approximating the power spectrum of an integrate-and-fire model driven by non-Poissonian shot noise. We demonstrated that this theory works surprisingly well and can capture the information-filtering effect seen in the spectral coherence function of the PSC in both the mean- and fluctuation-driven regimes.

We specifically chose to incorporate stochastic synapses in our two-stage model in order to reflect the variability in the effect of individual spikes seen in experiments (Lefort et al. 2009). We found, somewhat surprisingly, that the whitening of the input from the first stage has an information filtering effect on the encoding of the stimulus in the PSC output, independent of whether it is a CD. Hence, even when the PSC is in an integrator mode, if its synapses are strongly stochastic, the coherence can display a maximum around the firing rate of the population neurons. In CD mode, this leads to an even sharper peak in the PSC’s coherence, i.e., the information filtering effects of stochastic synapses and of coincidence detection compound.

Our analytical results rely on the assumption of a broadband Gaussian stimulus: the signal was a band-limited noise that had a flat power spectrum up to a cutoff frequency of $f_c=4$ (in multiples of the inverse membrane time constant of the population neurons). Enlarging $f_c$ would only improve the agreement with the theory because the latter assumes infinite bandwidth. Decreasing $f_c$ substantially would mean that we drive the two-stage system by a *colored* (temporally correlated) noise, which would already lead to different response properties at the single-cell level (see e.g. Brunel et al. (2001), Fourcaud-Trocmé et al. (2003), Moreno-Bote and Parga (2010), Lindner (2016), Droste and Lindner (2017b)). Exploring our two-stage transmission problem for both colored intrinsic noise and correlated input signals certainly represents a difficult but interesting subject for potential future investigations.

Another opportunity to generalize the studied problem lies in the connection topology of the system. The theory developed in the second part may be applied to scenarios with more than two stages of transmission or with recurrent connections among neurons of one stage. This will cover cases of signal transmission in other sensory areas, for instance in the visual system, where lateral connections play an important role in the first stage of processing.

## Figures and Tables

**Fig. 10:**
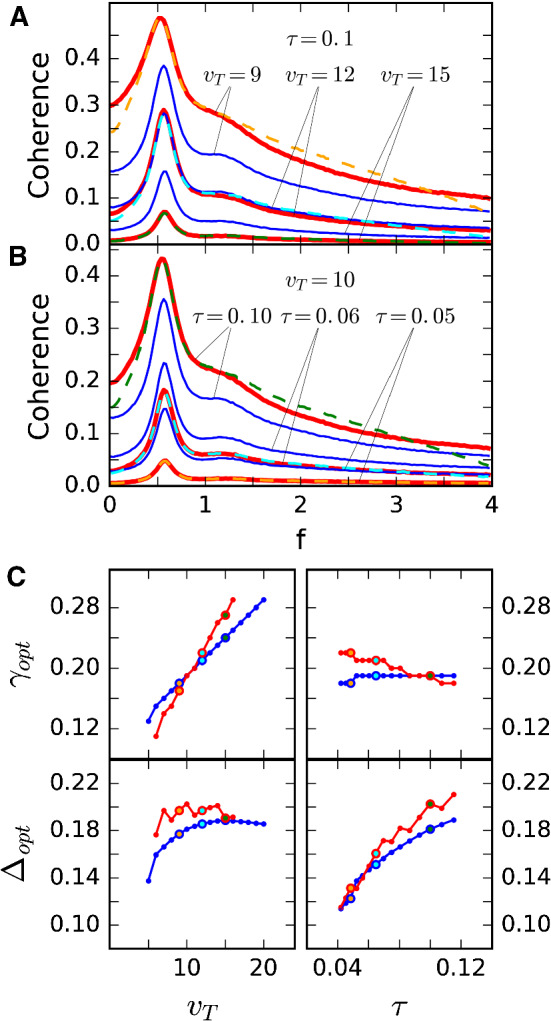
Extracted dependencies of $\gamma _\mathrm{{opt}}$ and $\varDelta _\mathrm{{opt}}$ on $\tau$ and $v_T$ for the case of constant amplitudes. Comparison of a selection of coherence functions of the CD in the constant regime (red) against those of the optimally matched SO (green, cyan and yellow). As a reference, coherence functions of the CD in the random synaptic amplitude regime are also shown (blue). In **a**, CD parameter $v_T$ is varied, while $\tau$ is held fixed, and vice versa in **b**. **c** Found relations between CD and SO parameters for a model with constant amplitudes (red) compared to results from Sect. (3) (blue) (color figure online)

**Fig. 11:**
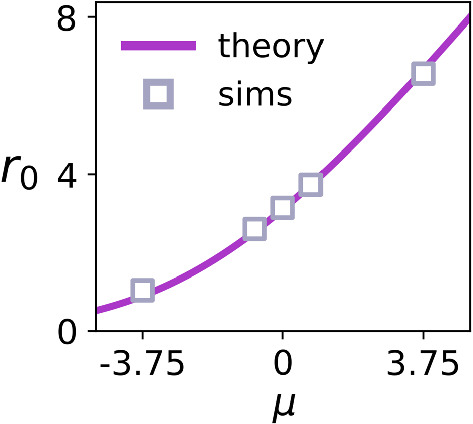
Steady-state rate of the PSC for different values of the external bias $\mu$. The magenta line shows the theory from Eq. (25). The gray boxes show the values calculated from the simulations in Fig. 7. Parameters are the same as in Fig. 7 (color figure online)

## Appendix

### Mapping synchrony encoding to PSC encoding for constant synaptic amplitudes

In this section, we repeat the procedure from Sect. (3), this time considering the case in which input to the PSC is weighted with constant synaptic amplitudes; as before, we focus on a parameter regime in which the PSC operates as a CD. Using this kind of input to the PSC, we would like to verify that the equivalence between the postsynaptic output and the partial synchrony does not hinge on a particular distribution of synaptic amplitudes. This is especially important because we saw that a randomization of synaptic amplitudes can contribute its own information filtering effect, regardless of spike synchrony. We also aim to characterize the dependencies between the parameters of the postsynaptic cell on the one hand and those of the partial synchronous output on the other hand.

Figure 10a, b displays a selection of PSC coherence functions (red) together with their optimally matched SO counterparts for different values of the postsynaptic cell’s threshold (A; time constant is fixed) and time constant (B; voltage threshold is fixed). We observe that also with constant synaptic amplitudes the SO can be tailored in a way that the profile of its coherence function closely mimics that of the PSC, at least in the proximity of the coherence peak. As a reference, we also show PSC coherence functions for the same parameters values but with random synaptic weights (blue), i.e., the case considered in the main part of the paper. Given the same parameter values for $\tau$ and $v_T$, coherence functions with constant amplitudes cover a larger range of magnitudes than those with random weights, which can be seen by comparing the red and blue curves in A or B. The additional noise provided to the PSC by the randomized amplitudes may reduce an already high coherence or boost a weak coherence (observed here at a high threshold), presumably by the effect of stochastic resonance (Gammaitoni et al. 1998; McDonnell and Abbott 2009).

Figure 10c shows the extracted relations between the PSC and SO parameters for both scenarios. In the scenario with constant synaptic weights (red), we observe that dependencies qualitatively similar to those from Sect. (3) (shown in blue for comparison) can be recovered. There are, however, a few noticeable differences. First, we see that the approximately linear relations between $\gamma _\text {opt}$ and $v_T$ and between $\varDelta _\text {opt}$ and $\tau$ are steeper than those observed in Sect. (3). This is a direct consequence of the fact that the PSC coherence functions from the scenario with constant amplitudes can assume a larger range of magnitudes as discussed above: with constant synaptic amplitudes, the SO parameters have to be changed over a broader range when fitting the PSC coherence in order to account for the larger change in magnitudes as $v_T$ or $\tau$ are varied. Secondly, the extracted relations are not as smooth as those discussed in Sect. (3), e.g., Fig. 10c lower left subplot. Increasing the size of the ensemble of spike trains and stimuli realizations from which all the spectral properties are estimated from $10^4$ to $10^5$ did not significantly reduce the observed variations, and we therefore conclude that they reflect some underlying fine structure in the parametric dependence. Thirdly, in contrast to the case with random amplitudes treated in the main text, there is a weak anticorrelation between the PSC parameter $\tau$ and SO parameter $\gamma _\mathrm{{opt}}$: an increase in the former leads to a decrease in the latter.

### Analytical expressions for firing rates, power spectra, and susceptibilities

For convenience, the theoretical spectral measures used in the text are reproduced below. In order to reduce clutter, angular frequencies ($\omega$) are written instead of temporal frequencies (*f*) as in the text.

#### Gaussian white noise

An LIF neuron with intrinsic Gaussian white noise has a stationary firing rate given by (Ricciardi 1977):

22 $$\begin{aligned} r_{\text {GN}} = \Bigg [ \tau _{\text {ref}} + \sqrt{\pi } \int _{\frac{\mu - v_T}{\sqrt{2D}}}^{\frac{\mu - v_R}{\sqrt{2D}}} e^{z^2} {{\,\mathrm{erfc}\,}}(z) dz \Bigg ]^{-1} \end{aligned}$$

and a power spectrum with the form (Lindner et al. 2002):

23 $$\begin{aligned} S_{\text {GN}}(\omega ) = r_{\text {GN}} \frac{ \Bigg | \mathcal {D}_{i\omega }\Big ( \frac{\mu - v_T}{\sqrt{D}} \Big ) \Bigg |^2 - e^{2\varDelta } \Bigg | \mathcal {D}_{i\omega }\Big ( \frac{\mu - v_R}{\sqrt{D}} \Big ) \Bigg |^2 }{ \Bigg | \mathcal {D}_{i\omega }\Big ( \frac{\mu - v_T}{\sqrt{D}} \Big ) - e^{\varDelta }e^{i \omega \tau _{\text {ref}}} \mathcal {D}_{i\omega }\Big ( \frac{\mu - v_R}{\sqrt{D}} \Big ) \Bigg |^2 }, \end{aligned}$$

where

$$\begin{aligned} \varDelta = \frac{v_R^2 - v_T^2 + 2 \mu (v_T - v_R)}{4D} \end{aligned}$$

and $D_{i\omega }(z)$ is the parabolic cylinder function (Abramowitz and Stegun 1970).

The susceptibility to a signal can be expressed as (Lindner and Schimansky-Geier 2001):

24 $$\begin{aligned} \chi _{\text {GN}}(\omega ) = \frac{r_{\text {GN}}}{\sqrt{D}} \frac{i \omega }{i \omega - 1} \frac{ \mathcal {D}_{i\omega - 1} \Big ( \frac{\mu - v_T}{\sqrt{D}} \Big ) - e^{\varDelta } \mathcal {D}_{i\omega - 1}\Big ( \frac{\mu - v_R}{\sqrt{D}} \Big ) }{ \mathcal {D}_{i\omega } \Big ( \frac{\mu - v_T}{\sqrt{D}} \Big ) - e^{\varDelta }e^{i \omega \tau _{\text {ref}}} \mathcal {D}_{i\omega } \Big ( \frac{\mu - v_R}{\sqrt{D}} \Big ) } \end{aligned}$$

(for the detailed calculation, see Lindner (2002) and for an equivalent expression, see Brunel et al. (2001)).

#### Shot noise

The rate given by Richardson and Swarbrick (2010) can be extended to include a bias $\mu$ as appears in our model by simply shifting the voltage, i.e., subtracting the bias value from the threshold and reset voltages:

25 $$\begin{aligned} r_{\text {SN}} =\Bigg [ \tau _m \int _{0}^{1/\left\langle a_k \right\rangle } \frac{1}{s Z_0(s)} \Big ( \frac{e^{s (v_T - \mu )}}{1 - \left\langle a_k \right\rangle s} - e^{s (v_R - \mu )} \Big ) ds \Bigg ]^{-1} \end{aligned}$$

where $\left\langle a_k \right\rangle$ is the average synaptic weight and the generating function for the subthreshold-voltage moments,

26 $$\begin{aligned} Z_0(s) = (1 - \left\langle a_k \right\rangle s)^{-\tau _m R_{\text {pop}}} , \end{aligned}$$

receives the total population rate $R_{\text {pop}}=Nr_{\text {GN}}$.

Figure 11 plots Eq. (25) along with the simulated rates at the values of $\mu$ used in Fig. 7. The theory generally captures the trend shown by the simulations, especially for low bias magnitudes.

The power spectrum in this case has the form (Droste 2015; Droste and Lindner 2017a) (an equivalent theory can be found in Richardson and Swarbrick (2010)):

27 $$\begin{aligned} S_{\text {SN}}(\omega ) = r_{\text {SN}} \frac{ \Big | e^{- i \omega \tau _{\text {ref}}} \mathcal {F}(v_T, \omega ) \Big |^2 - \Big | \frac{R_{\text {pop}}}{R_{\text {pop}}- i\omega } \mathcal {G}(v_R, \omega ) \Big |^2 }{ \Big | e^{- i \omega \tau _{\text {ref}}} \mathcal {F}(v_T, \omega ) - \frac{R_{\text {pop}}}{R_{\text {pop}} - i\omega } \mathcal {G}(v_R, \omega ) \Big |^2 }, \end{aligned}$$

where $\mathcal {F}(v, \omega )$ and $\mathcal {G}(v, \omega )$ are the confluent hypergeometric functions (Abramowitz and Stegun 1970):

28 $$\begin{aligned}&\mathcal {F}(v, \omega ) {:=} {}_{1}F_{1}\Big (- i \omega \tau _m; (R_{\text {pop}} - i \omega ) \tau _m; \frac{v - \mu }{\left\langle a_k \right\rangle }\Big ),\end{aligned}$$

29 $$\begin{aligned}&\mathcal {G}(v, \omega ) {:=} {}_{1}F_{1}\Big (- i \omega \tau _m; 1 + (R_{\text {pop}} - i \omega ) \tau _m; \frac{v - \mu }{\left\langle a_k \right\rangle }\Big ). \end{aligned}$$

The susceptibility is given by (Droste 2015; Droste and Lindner 2017a) (an equivalent theory, derived earlier, can be found in Richardson and Swarbrick (2010)):

30 $$\begin{aligned} \chi _{\text {SN}}(\omega ) = \frac{ \int _{v_R}^{v_T} P_0(v) \Big [ (R_{\text {pop}} - i\omega ) \mathcal {F}(v, \omega ) - R_{\text {pop}} \mathcal {G}(v, \omega ) \Big ] dv }{ (R_{\text {pop}} - i\omega ) \mathcal {F}(v_T, \omega ) - R_{\text {pop}} e^{i \omega \tau _{\text {ref}}} \mathcal {G}(v_R, \omega ) } \end{aligned}$$

with the stationary probability density

31 $$\begin{aligned} P_0(v) = \frac{\tau _m r_{\text {SN}}}{\left\langle a_k \right\rangle } \frac{e^{-\phi (v)}}{\mu - v} \int _{v_T}^{v} e^{\phi (x)}\mathrm{d}x \end{aligned}$$

and

32 $$\begin{aligned} \phi (v) = \frac{v}{\left\langle a_k \right\rangle } - \tau _m R_{\text {pop}} \ln (|\mu - v|) \end{aligned}$$

for $$\mu > v_R$$.

### Spectral measures for the population spike output with constant or random amplitudes

The PSC receives the population output *y*(*t*) as an ensemble of spike trains and a weight $a_k$ is assigned to each spike, giving a total input *I*(*t*) to the PSC:

33 $$\begin{aligned} I(t) = \sum _{k} a_k \delta (t - t_k)=\sum _i \hat{y}_i(t) \end{aligned}$$

here, the sum with index *i* runs over both neurons and spike times; in the second step, we have expressed the input by a sum of each individual neuron’s output spike train, endowed with its specific weights $\hat{y}_i(t)=\sum _{\ell } a_{i, \ell } \delta (t-t_{i,\ell })$.

We will study two cases: constant amplitudes ($a_k\equiv 1$) and random amplitudes, each independently drawn from an exponential distribution, i.e.,

34 $$\begin{aligned} Prob(a<a_k<a+da)=\exp (-a) da \end{aligned}$$

implying that the mean value is the same as for the constant amplitudes ($\left\langle a_k \right\rangle =1$) and that the standard deviation of the amplitude is equal to its mean. In other words, the ratio of standard deviation to mean, the coefficient of variation, $C_{V,a}=1$. In the case of constant (non-random) amplitudes, this coefficient vanishes ($C_{V,a}=0$).

For general mean value and coefficient of variation of the amplitude, we want to calculate i) the cross-spectrum between *I*(*t*) and the signal and ii) the power spectrum of the weighted sum *I*(*t*). The first task is simple. Because the amplitudes are unrelated to the signal and to the intrinsic noise in the population dynamics that renders the spike times stochastic, we can separate the average over the $a_k$ (average $\left\langle \right\rangle _a$), the intrinsic noise (average $\left\langle \right\rangle _\xi$) and the signal (average $\left\langle \right\rangle _s$) and obtain for the cross-correlation function

35 $$\begin{aligned} \left\langle I(t) s(t+\tau ) \right\rangle _{a,\xi ,s}= & {} \left\langle \left\langle I(t) \right\rangle _a s(t+\tau ) \right\rangle _{\xi ,s}\nonumber \\= & {} \left\langle \sum _{k} \left\langle a_k \right\rangle _a \delta (t - t_k) s(t + \tau ) \right\rangle _{\xi ,s}\nonumber \\= & {} \left\langle a_k \right\rangle _a \left\langle \sum _i y_i(t)s(t+\tau ) \right\rangle _{\xi ,s}\nonumber \\= & {} \left\langle a_k \right\rangle _a \left\langle y(t)s(t+\tau ) \right\rangle _{\xi ,s}, \end{aligned}$$

where *y*(*t*) is the population output, i.e., the sum of the *N* spike trains $y_i(t)$ of the population neurons, whose correlation is obviously described by the linear-response function, or by the susceptibility in the Fourier domain, which yields (equivalent to Eq. (19))

36 $$\begin{aligned} \left\langle \tilde{I} \tilde{s}^* \right\rangle _{a,\xi ,s}=N\left\langle a_k \right\rangle _a \chi _{GN} \left\langle \tilde{s}\tilde{s}^* \right\rangle . \end{aligned}$$

Next, we consider the autocorrelation function of *I*(*t*) and split it into terms for the single (amplitude-modified) spike trains $\hat{y}_i(t)$ (omitting the ensemble average indices)

37 $$\begin{aligned}&\left\langle I(t) I(t+\tau ) \right\rangle = \left\langle \sum \hat{y}_i(t)\sum \hat{y}_j(t+\tau ) \right\rangle \nonumber \\&= N \big [\left\langle \hat{y}_1(t) \hat{y}_1(t+\tau ) \right\rangle +(N-1)\left\langle \hat{y}_1(t) \hat{y}_2(t+\tau ) \right\rangle \big ] \end{aligned}$$

where we arbitrarily chose $\hat{y}_1(t)$ and $\hat{y}_2(t)$ as statistical representatives to express the autocorrelation and cross-correlation terms. For the latter factor, an average over the amplitudes is straightforward and gives

38 $$\begin{aligned} \left\langle \hat{y}_1(t) \hat{y}_2(t+\tau ) \right\rangle _{a,\xi ,s}=\left\langle a_k \right\rangle _a^2 \left\langle y_1(t) y_2(t+\tau ) \right\rangle _{\xi ,s} \end{aligned}$$

and is just a scaled version of the spike-train correlation function, which is entirely due to the common stimulus. In the Fourier domain, this cross-correlation term will thus result in

39 $$\begin{aligned} \left\langle a_k \right\rangle _a^2 |\chi _{GN}|^2 S_s. \end{aligned}$$

For the autocorrelation term, we write the averaged product of amplitudes as follows

40 $$\begin{aligned} \left\langle a_k a_\ell \right\rangle _a=\delta _{k,\ell } \left\langle a_k^2 \right\rangle _a+(1-\delta _{k,\ell }) \left\langle a_k \right\rangle _a^2, \end{aligned}$$

where we have used the independence of the $a_k$. Exploiting this relation in the autocorrelation function, we obtain (dropping the indices for $\hat{y}_1$ and the synaptic ensemble averages)

41 $$\begin{aligned} \left\langle \hat{y}(t)\hat{y}(t+\tau ) \right\rangle= & {} (\left\langle a_k^2 \right\rangle -\left\langle a_k \right\rangle ^2)\sum _k \delta (t-t_k)\delta (t+\tau -t_k)\nonumber \\&+\left\langle a_k \right\rangle ^2\sum _{k,\ell } \delta (t-t_k)\delta (t+\tau -t_\ell )\nonumber \\= & {} \left\langle a_k \right\rangle ^2\left( C_{V,a}^2r_0^\mathrm{{POP}}\delta (\tau )+\left\langle x(t)x(t+\tau ) \right\rangle \right) \end{aligned}$$

where we used that $\delta (t-t_k)\delta (t+\tau -t_k)=\delta (t-t_k)\delta (\tau )$ and $\left\langle \sum _k \delta (t-t_k) \right\rangle =r_0^\mathrm{{POP}}$. The Fourier transform of this expression, along with the usual linear response approximation for the spike-train power spectrum as well as Eqs. (39) and (37), leads to the final expression for the power spectrum Eq. (20).
